# Role of predisposition, injury, response and organ failure in the prognosis of patients with acute-on-chronic liver failure: a prospective cohort study

**DOI:** 10.1186/cc11882

**Published:** 2012-11-27

**Authors:** Rajiv Jalan, Vanessa Stadlbauer, Sambit Sen, Lisa Cheshire, Yu-Mei Chang, Rajeshwar P Mookerjee

**Affiliations:** 1 The Liver Failure Group, Institute of Liver and Digestive Health, UCL Medical School, Royal Free Hospital, Rowland Hill Street, London NW3 2PF, UK; 2 Department of Internal Medicine, Medical University of Graz, Auenbruggerplatz 15, 8036 Graz, Austria; 3 Research Support Office, Royal Veterinary College, University of London, Royal College Street, London NW1 0TU, UK

## Abstract

**Introduction:**

Acute deterioration of cirrhosis is associated with high mortality rates particularly in the patients who develop organ failure (OF), a condition that is referred to as acute-on-chronic liver failure (ACLF), which is currently not completely defined. This study aimed to determine the role of predisposing factors, the nature of the precipitating illness and inflammatory response in the progression to OF according to the PIRO (predisposition, injury, response, organ failure) concept to define the risk of in-hospital mortality.

**Methods:**

A total of 477 patients admitted with acute deterioration of cirrhosis following a defined precipitant over a 5.5-year period were prospectively studied. Baseline clinical, demographic and biochemical data were recorded for all patients and extended serial data from the group that progressed to OF were analysed to define the role of PIRO in determining in-hospital mortality.

**Results:**

One hundred and fifty-nine (33%) patients developed OF, of whom 93 patients died (58%) compared with 25/318 (8%) deaths in the non-OF group (*P *< 0.0001). Progression to OF was associated with more severe underlying liver disease and inflammation. In the OF group, previous hospitalisation (P of PIRO); severity of inflammation and lack of its resolution (R of PIRO); and severity of organ failure (O of PIRO) were associated with significantly greater risk of death. In the patients who recovered from OF, mortality at three years was almost universal.

**Conclusions:**

The results of this prospective study shows that the occurrence of OF alters the natural history of cirrhosis. A classification based on the PIRO concept may allow categorization of patients into distinct pathophysiologic and prognostic groups and allow a multidimensional definition of ACLF.

## Introduction

WHO projections estimate that liver cirrhosis will be the ninth most common cause of death in the western world by 2015. The cost of management of patients who require hospital admission with acute deterioration in the US approximates $13 billion [1]. In a significant proportion of such patients, death is related to multiple organ failure, which, when manifest, carries a high mortality rate. Currently, these patients do not have priority for listing for liver transplantation and often such patients are considered to be too ill for transplantation [2]. Conceptually, this multiorgan failure can occur either as a slow gradually progressive decompensation culminating in multiple organ failure or it can occur as a result of a precipitating illness, which is often of non-hepatic origin [3] on the background of cirrhosis in a previously stable cirrhotic patient, who may or may not have a history of decompensation. While both entities can lead to various features of multiorgan failure the underlying mechanisms of decompensation are probably quite different and, therefore, hypothetically the clinical outcome is likely to be different. This latter condition has been referred to in the literature as having acute-on-chronic liver failure (ACLF) [4].

In order to clinically describe the group of patients referred to as ACLF, we adopted the definition that these patients would have 'acute deterioration in liver function over a short period (up to four weeks)' 'associated with a precipitating event' 'in patients with previously well-compensated liver disease' 'characterised by organ failure' [5]. We also hypothesised that from the clinical standpoint the most important difference between the two entities is the potentially reversible nature of ACLF if precipitants could be controlled.

Several studies have addressed the outcome of patients with liver cirrhosis and organ failure. The requirement for ICU admission in patients with cirrhosis was associated with high mortality rates ranging from 40 to 90%. Wehler *et al. *[6] assessed the impact of organ failure and showed that the presence of organ failure with the Sepsis Organ Failure Assessment (SOFA) score of 9 or greater was associated with a short-term mortality of about 90%. However, this study did not specifically address the question if there is a difference between patients with acute deterioration compared to those with end-stage liver disease. Identification of patients at risk of progressing to multiorgan failure would help substantially in prioritising patients for early intensive therapy, transfer to specialist tertiary referral units, liver support and listing for transplantation.

We hypothesised that a concept similar to that used in determining outcomes of sepsis [7] may be useful in defining outcomes in patients who develop organ failure, the PIRO concept; Predisposition (P); Injury (I) by the nature of the insult leading to decompensation; Response (R) to this insult and the development of organ failure (O) are the four most important factors determining outcome. The aim of this study was to define the natural history of patients with acute deterioration of cirrhosis without existing organ failure other than liver cirrhosis that were admitted to the hospital and investigate the factors leading to occurrence of organ failure in the hospital and death.

In this study, a group of patients with liver cirrhosis that were admitted to a single unit over a 5.5-year period and managed according to pre-defined protocol were recruited. The main questions the prospective study was set up to answer were whether the outcome of patients with acute decompensation of cirrhosis due to a defined precipitant who progress to single organ failure in comparison to those who do not develop organ failure was different and determine the factors associated with mortality of patients with organ failure. We also determined whether a previous episode of decompensation influences outcome and whether the inflammatory response to the precipitating illness was associated with poor outcome.

### Materials and methods

Consecutive patients with liver cirrhosis admitted to the University College London Hospitals (UCLH) with decompensated liver cirrhosis between July 2000 and January 2006 were evaluated for entry into the study. This prospective single-centre study was performed on a group of patients that were in a pre-screen log for inclusion into a randomised study of liver support device (treatment of disturbed inter-organ metabolism in decompensated cirrhosis using molecular adsorbent recirculating system (MARS)). This study was approved by the UCLH Ethics Committee and conducted according to the Declaration of Helsinki. As the patients were a part of a pre-screening log, no additional consent was required.

### Recruitment and study enrolment

All patients of any age with an acute clinical deterioration of presumed cirrhosis (elevated bilirubin > 85 μmol/L, or/and increasing ascites or/and hepatic encephalopathy < grade 2) related to a clear precipitating event (infection, bleeding, alcoholic hepatitis, exposure to hepatotoxins) were included and data were collected retrospectively and prospectively. The diagnosis of cirrhosis was confirmed either by liver biopsy or by clinical signs (signs of portal hypertension and imaging concordant with cirrhosis). The patients were included if they failed to show signs of improvement in their presenting complaints or biochemistry, 48 hours after admission and following correction of precipitating illnesses.

Exclusion criteria: admission for reasons other than decompensation of cirrhosis (other co-morbid diseases, especially established cardiovascular or renal disease); presence of organ failure (as defined later), malignancy (extra-hepatic or a hepatocellular carcinoma); patients who have undergone major surgery (for example liver resection) or have unsolved surgical problems; pregnancy.

### Study design and management

All patients were followed up until the end of the study, death or liver transplantation. Survival data for the surviving patients were available for one year after the end of the study (end 2006). Patients were managed according to a pre-defined standard of care and all patients that were included had the potential to be supported with full intensive care if required. Briefly, the standard of care for the patients included the following:

1. Nutritional support: enteral feeding with a calorie intake > 30 Kcal/Kg/day with additional vitamin supplementation especially in alcoholic patients.

2. Evidence of suspected or culture-positive infection: intravenous antibiotics covering gram-positive and -negative organisms in accordance with local institution microbiology policies.

3. Re-accumulation of ascites: sodium chloride restriction (≤ 100 mmol/day) and therapeutic paracentesis with albumin replacement (8 g/litre of ascites removed).

4. New onset renal impairment: fluid challenge with colloid and crystalloid, and if deemed to have developed hepatorenal syndrome, managed with terlipressin 0.5 to 2 mg intravenously, up to six times daily concurrent with infusion of 60 g salt-poor albumin.

5. Progressive organ failure (defined below): full intensive care support including haemofiltration or/and ventilation, as indicated.

### Definitions

1. Definition of organ failure *was *based on modification of the SOFA score:

a. Circulation: need for inotropes to maintain mean arterial pressure greater than 65 mmHg (modified SOFA: 3 or 4); the use of terlipressin for hepatorenal syndrome was not considered as inotropic support but a specific treatment hepatorenal syndrome.

b. Renal and acid-base disturbances: requirement for haemofiltration to correct acidosis and/or oligo-anuria with serum creatinine > 221 umol/L following correction of any intravascular volume deficit and with no evidence of pre-existing renal failure (modified SOFA: 2 or more).

c. Inadequate oxygenation: PO2/FIO2 > 200 of SpO2/FiO2 < 214 or requirement for mechanical ventilation to maintain an arterial partial pressure of oxygen > 10 kPA (modified SOFA: 3 or 4).

d. Severe encephalopathy: grade 3 and 4 and/or need for mechanical ventilation for airway compromise (modified SOFA: 4).

e. Severe progressive hyperbilirubinaemia: a progressive increase in bilirubin to > 340 umol/dl (modified SOFA: 4).

f. Severe coagulopathy: INR > 2.5 or platelet count ≤ 20,000 (modified SOFA: 4).

2. Systemic Inflammatory Response Syndrome (SIRS) was defined by the presence of two or more of the following: temperature > 38°C or < 36°C; heart rate > 90 beats/min; respiratory rate > 20 per min or PaCO2 < 32 mmHg; white blood cells > 12,000 cells/mm^3 ^or < 4000 cells/mm^3^.

3. Infection-positive cultures of blood, ascites, urine, sputum or wounds and/or clinical findings suggestive for infections (chest X-ray). A new (nosocomial) infection was defined as an infection that occurred more than 48 hours after admission or 48 hours after clearance of an existing infection. The diagnosis of infection in this study was based on standard routine clinical procedures. It is not possible to exclude the rate of diagnosis of bacterial infection, which would have been higher if more rigorous testing was used. In order to ensure reproducibility of diagnosis, the use of antibiotics in this group of patients for either confirmed or presumed diagnosis of bacterial infection is always done in close discussion with a designated microbiologist.

### Data collection

Baseline data and mortality were recorded for all patients and an extended dataset including serial data at days 0 (onset of organ failure), 3 and 7 regarding clinical and demographic variables were determined in the organ failure group (Table S2 in Additional file 1). Child-Pugh score and Model for End-Stage Liver Disease (MELD) score evaluated severity of liver disease at days 0, 3 and 7. Acute Physiology, Age and Chronic Health Evaluation (APACHE) II score was used for determination of illness severity and the SOFA score for grading of organ dysfunction at days 0, 3, and 7. The presence or absence of SIRS was recorded at days 0, 3 and 7 and the number of failing organs during the first week on an Intensive Care Unit (ICU) was counted according to the above definition. SOFA score was calculated as published with a few modifications.

APACHE II and SOFA scores were calculated after the onset of organ failure. Some patients were not on an ICU at the onset of organ failure. Since we do not have data on the reason why patients were not admitted to ICU, we calculated these scores for all patients with organ failure [6,8-12].

### Statistical methods

Comparison of demographic and clinical parameters between groups was performed using independent variable *t *test or Mann-Whitney *U *tests for continuous variables and chi-square tests for categorical variables. More than two groups were compared using two-way ANOVA. Survival curves of two groups were compared graphically using the method of Kaplan-Meier, counting death as event. The equality of the dichotomised groups was tested via log-rank test. The discrimination ability of single parameters or scores to predict outcome of patients was evaluated by calculating the area under the receiver operating characteristics curve (AUROC). The Younden Index was used to select the best cutoff point, at which sensitivity, specificity, positive predictive value and negative predictive value were calculated. To identify factors that were independently associated with outcome (30-day mortality, censored at time of transplantation), univariate and multivariable logistic regression analysis were performed. Risk factors with a significance of *P *< 0.10 in the univariate analysis were entered manually into the multivariable model using stepwise selection. The scores (SOFA, APACHE II, Child and MELD) were not incorporated into the multivariable analysis. The statistical significance level was set as *P *< 0.05. Continuous variables were summarised as mean ± SEM, and categorical variables were summarised as proportions. Results from logistic regression were given as odds ratio (OR) and 95% confidence intervals (CI). All analyses were carried out using SPSS statistics software version 17 (SPSS Inc., Chicago, IL, USA).

## Results

### The study cohort

**Figure 1:**
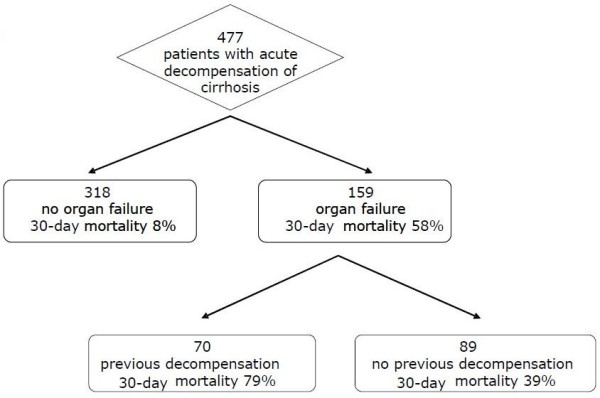
**Study flow and summary of outcomes**.

**Figure 2:**
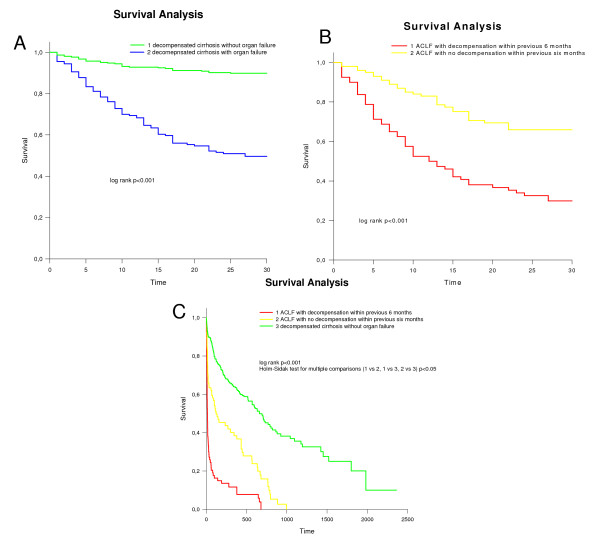
**Survival analyses of included patients**. **(a) **Thirty-day mortality the patients with and without organ failure (log-rank test: *P *< 0.001). The analyses started from the day of onset of organ failure. **(b) **Thirty-day mortality of patients with organ failure divided according to whether they had a previous hospital admission with decompensated cirrhosis within the previous six months (log-rank test: *P *< 0.001). **(c) **Long-term outcome of patients with acute deterioration of cirrhosis that did not develop organ failure compared with the patients with organ failure. The organ failure group is further subdivided into those who required hospital admission with an episode of decompensation within the previous six months and those that did not (log-rank test: *P *< 0.001).

In total 497 inpatients admitted for acute deterioration of cirrhosis were recruited into the study (Table 1). Twenty patients were excluded from the final analysis because of lack of data or not fulfilling study criteria. The study group, therefore, consisted of 477 patients. Three hundred and eighteen patients did not develop organ failure (non-organ failure group from here). (Figure 1) Thirty-day mortality in the non-organ failure group was 8% (Figure 2a) and the one-year mortality was 42%. A total of 159 patients developed organ failure (organ failure group; mean age 52 ± 0.7, 75% male, aetiology: alcohol 75%, other 11%, both 14%, Table 1). Thirty-day mortality of the 159 patients who developed organ failure was 58%. (Figure 2a, Table 1). Forty-two patients (26%) were not admitted to ICU.

**Table 1:** Description of the whole study cohort at inclusion.

	All (*n *= 477)	Non-organ failure (*n *= 318)	Organ failure (*n *= 159)
***Predisposition***			
Age	53.1 ± 0.5	53.6 ± 0.72	52.0 ± 0.7
Gender (M/F)	322/153	203/113	119/40*
Aetiology of cirrhosis	354 (74%)	239 (75%)	
Alcohol	92 (19%)	52 (16%)	115 (72%)
Hepatitis B/C	31 (7%)	27 (8%)	40 (24%)
Others			4 (3%)
INR	1.70 ± 0.03	1.67 ± 0.05	1.76 ± 0.04
Bilirubin (umol/l)	156.9 ± 8.5	98.9 ± 7.2	273.5 ± 17.9***
Albumin (g/L)	28.7 ± 0.6	30.0 ± 0.8	25.8 ± 0.5***
Creatinine (umol/L)	110.4 ± 4.0	93.4 ± 1.2	146.8 ± 11.7***
Child-Pugh score	10.7 ± 0.1	10.3 ± 0.4	11.2 ± 0.2***
MELD score	12.3 ± 0.4	10.5 ± 0.1	16.4 ± 0.8***
ALT (U/L)			69.4 ± 8.0
Haemoglobin (g/dL)			10.4 ± 0.8
Platelets (x10^9^/L)			111.5 ± 6.1
Heart rate (/min)			96 ± 1.6
Body temperature (°C)			36.8 ± 0.1
***Previous decompensation***			
Ascites			52 (32)
Variceal bleed			36 (22)
Hepatic encephalopathy			22 (14)
Jaundice			47 (29)
***Precipitating event (I)***			
Infection		171 (54)	76 (47)
Variceal bleed		108 (32)	46 (28)
Alcohol binge		127 (40)	76 (47)
Others		48 (16)	25 (16)
***Response (R)***			
SIRS		-	71 (45)
CRP		31.2 ± 5.4	56.4 ± 3.3***
Infection		-	50 (31)
***Organ failure (O)***		None (0)	All (159)
APACHE II		-	13.5 ± 0.6
SOFA		-	8.3 ± 0.8
MARS therapy		0 (0)	43 (27)
**In-hospital mortality**	118/477 (25)	25/318 (8)	93/159 (58)***

Data expressed as mean (SEM). *P < 0.05 compared to non-organ failure group. **P < 0.01 compared to non-organ failure group. ***P < 0.001 compared to non-organ failure group. ALT, alanine aminotranferase; APACHE II, Acute Physiology, Age and Chronic Health Evaluation; CRP, C-reactive protein; INR, international normalised ratio; MARS, molecular adsorbent recirculating system; MELD, Model for End-Stage Liver Disease; SIRS, Systemic Inflammatory Response Syndrome; SOFA, Sepsis Organ Failure Assessment.

### Factors associated with mortality in the organ failure group according to PIRO classification

#### Predisposition (P)

Age, gender and aetiology of liver disease as possible predisposing factors were not different between survivors and non-survivors. Although more men developed organ failure (37% versus 26%, *P *= 0.02), there was no difference in mortality between men and women in the organ failure (OF) group. For baseline biochemistry and comparisons between survivors and non-survivors see Table 2. Non-survivors had significantly higher levels of creatinine, bilirubin, prothrombin time (PT), international normalised ratio (INR), and activated partial thromboplastin time (aPTT) and lower albumin levels compared to survivors at baseline. Bilirubin and INR were also independent predictors of mortality in the multivariable analysis. Child and MELD score were predictive for mortality on univariate analysis (Table 3) but these scores were not included into the multivariable analysis.

**Table 2:** Differences between survivors and non-survivors at the time first organ failure is diagnosed.

	Survivors (*n *= 66)	Non-survivors (*n *= 93)
**Predisposition**		
Age	51.1 ± 1.1	52.5 ± 0.9
Gender (M/F)	47/19	72/21
Aetiology alcohol N (%)	45 (68)	70 (75)
PT (sec)	15.9 ± 0.4***	20.7 ± 0.6
INR	1.54 ± 0.04**	1.91 ± 0.06
aPTT (sec)	49.9 ± 2.0***	65.5 ± 3.4
Bilirubin (umol/L)	190.6 ± 20.0***	331.2 ± 25.4
Albumin (g/L)	27.3 ± 0.9**	24.8 ± 0.7
Creatinine (umol/L)	114.8 ± 13.3***	170.5 ± 16.9
Child	10.1 ± 0.2***	12.1 ± 0.2
MELD	12.0 ± 1.0***	19.6 ± 1.1
Hospitalisation within last 6 months N (%)	15 (23)	55 (59)
**Injury**		
Infection N (%)	28 (42)	54 (58)
Variceal bleed N (%)	22 (33)	23 (24)
Alcohol binge N (%)	40 (60)	47 (51)
Dehydration N (%)	6 (9)	15 (16)
Drugs N (%)	2 (3)	1 (1)
**Response**		
SIRS N (%)	25 (38)	46 (49)
Infection N (%)	12 (18)**	38 (41)
**Organ failure**		
Inotrope N (%)	3 (5)***	44 (47)
Renal failure N (%)	12 (18)***	53 (57)
Haemofiltration N (%)	7 (11)***	38 (41)
Mechanical ventilation N (%)	10 (15)***	45 (48)
Hepatic encephalopathy	39 (59)	52 (56)
Hyperbilirubinemia > 340 umol/l	10 (15)***	38 (41)
Severe coagulopathy	1 (2)**	15 (18)
APACHEII	10.9 ± 0.8*	15.1 ± 0.8
SOFA	6.7 ± 0.3**	9.4 ± 0.3

*P < 0.05; **P < 0.005; ***P < 0.001; data expressed as mean (SEM). APACHE II, Acute Physiology, Age and Chronic Health Evaluation; aPTT, activated partial thromboplastin time; INR, international normalised ratio; MELD, Model for End-Stage Liver Disease; PT, prothrombin time; SIRS, Systemic Inflammatory Response Syndrome; SOFA, Sepsis Organ Failure Assessment.

**Table 3:** Univariate and multivariable logistic regression analysis of mortality at the time first organ failure is diagnosed.

		Univariate		Multivariable	
		
Risk factor	N	Crude OR (95% CI)	*P *value	Adjusted OR (95% CI)	*P *value
**Predisposition**					
Age	159	1.02 (0.98, 1.05)	0.324		
Gender (M/F)	159	0.72 (0.35, 1.48)	0.375		
Aetiology alcohol	159	1.42 (0.71, 2.86)	0.325		
PT (sec)	128	1.44 (1.23, 1.68)	< 0.0001		
INR (per unit)	159	8.94 (3.12, 25.66)	< 0.0001	6.43 (1.82, 22.71)	0.004
aPTT (sec)	132	1.04 (1.01, 1.06)	0.001		
Bilirubin (per 10 umol/L)	158	1.03 (1.02, 1.05)	< 0.0001	1.04 (1.02, 1.07)	0.001
Albumin (g/L)	145	0.94 (0.89, 0.99)	0.027		
Creatinine (per 10 umol/L)	148	1.04 (1.00, 1.07)	0.028		
Child score	152	1.91 (1.51, 2.41)	< 0.0001		
MELD score	146	1.09 (1.05, 1.14)	< 0.0001		
Hospitalisation within last 6 months	159	4.92 (2.42, 10.00)	< 0.0001	4.97 (1.89, 13.09)	0.001
**Injury**					
Infection	159	1.88 (0.99, 3.56)	0.053		
Variceal bleed	159	0.66 (0.33, 1.32)	0.237		
Alcohol binge	159	0.66 (0.35, 1.26)	0.210		
Dehydration	159	1.92 (0.70, 5.25)	0.202		
Drugs	159	0.35 (0.03, 3.92)	0.393		
**Response**					
SIRS score	158	1.38 (1.00, 1.91)	0.051		
Infection	152	3.05 (1.43, 6.49)	0.004		
**Organ failure**					
Inotrope	152	18.81 (5.49, 64.45)	< 0.0001	14.70 (3.18, 68.03)	0.001
Renal failure	153	6.09 (2.86, 12.97)	< 0.0001	3.46 (1.18, 10.12)	0.023
Haemofiltration	152	5.96 (2.45, 14.53)	< 0.0001		
Mechanical ventilation	152	5.42 (2.45, 11.98)	< 0.0001		
Hepatic encephalopathy	159	0.88 (0.46, 1.66)	0.690		
Hyperbilirubinemia > 340 umol/l	159	3.87 (1.76, 8.52)	0.001		
Severe coagulopathy	144	13.24 (1.70, 103.20)	0.014		
APACHE II score	114	1.12 (1.04, 1.20)	0.002		
SOFA score	153	1.51 (1.29, 1.77)	< 0.0001		

Due to different amount of missing data and correlations among predictors, stepwise selection was done manually to build the multivariable model. 151 observations were included in the final multivariable analysis. The scores (Child, MELD, SOFA, APACHE II) were not incorporated into the multivariable analysis. APACHE II, Acute Physiology, Age and Chronic Health Evaluation; aPTT, activated partial thromboplastin time; CI, confidence interval; INR, international normalised ratio; MELD, Model for End-Stage Liver Disease; OR, odds ratio; PT, prothrombin time; SIRS, Systemic Inflammatory Response Syndrome; SOFA, Sepsis Organ Failure Assessment.

Seventy of the 159 patients had at least one episode of decompensation requiring hospital admission within the previous six months. Thirty-day mortality of patients with previous decompensation was 79% whereas 39% of patients without previous decompensation within the previous six months died in the same period of time (*P *< 0.001, Figure 2b). We analysed the predictive utility of Child and MELD in the two groups using AUROC. Mortality in patients with previous decompensation was predicted at lower MELD cutoff scores compared with the group without previous decompensation (MELD 9.2 vs 10.7; Table S1 in Additional file 1).

Mortality of the patients who recovered from organ failure in the long-term was significantly worse than in the patients who did not develop organ failure. In patients with previous decompensation who went on to develop organ failure most of the patients died within one year (85.5%). None of the patients were alive beyond three years (*P *< 0.001). In patients who did not develop organ failure, long-term mortality was dictated by the severity of their underlying liver disease (Figure 2c).

Comparing patients with and without previous decompensation at baseline showed that patients did not differ significantly in all variables except for a higher aPTT and a higher baseline SOFA score in the group with previous decompensation. There was no difference in precipitating events for decompensation in patients with or without previous decompensation and there was also no difference in the development of new complications between these two groups (Table S2, S3 in Additional file 1). Previous decompensation within the last six months was an independent predictor of mortality in multivariable analysis with an adjusted OR of 4.97. Twenty-three percent of survivors, but 59% of non-survivors had a previous decompensation (*P *< 0.0001).

#### Injury (I)

The commonest precipitating illness leading to hospital admission was infection (47%), which was almost equally split between spontaneous bacterial peritonitis (54%) and other infection (chest: 19%; UTI: 12%; infected leg ulcer: 11%; others: 4%). Alcohol binge formed the next largest group amounting to 47% as well. Variceal bleeding accounted for about 28% of cases. More than one precipitating event could be present. There were no patients included in the study who did not have a defined precipitating illness. None of these precipitating factors was able to distinguish between survivors and non-survivors.

#### Systemic inflammatory response and infection (R)

**Figure 3:**
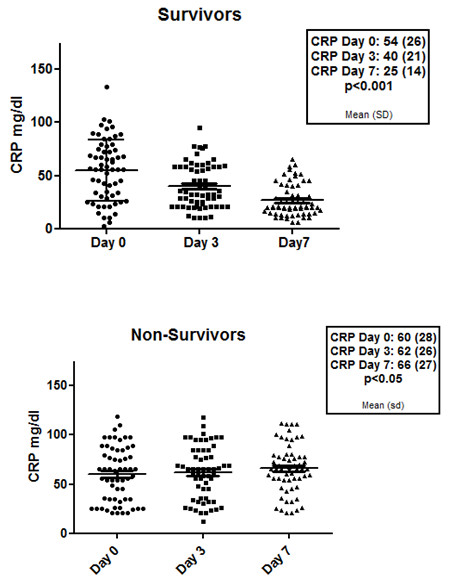
**Changes in C-reactive protein (CRP) (mean (SD)) over the first seven days in the patients that survived compared with the patients who died**. The data show that there was a significant reduction in CRP in the survivors (ANOVA: *P *< 0.001) whereas the CRP increased significantly in the non-survivors (ANOVA: *P *< 0.05).

SIRS occurred independently of infection. SIRS occurred in 42% of patients with organ failure and tended to be more frequent in non-survivors (46% vs 25%, *P *= 0.051). SIRS was not associated with the trigger infection. Although baseline C-reactive protein (CRP) was marginally higher in the non-survivors, the most important difference between the patients who survived was in their ability of the patients to resolve inflammation indicated by their ability to reduce CRP compared with the group who died (Figure 3a and 3b). Twenty-four percent of all patients with organ failure developed a new infection during ICU stay and the development of a new infection was associated with an increased mortality (74.1% vs 44.9%, *P *< 0.001). No statistically significant interactions were detected between the presence of SIRS and infection (*P *= 0.30).

#### Organ failure

The development of each new organ failure (see definition in Methods section) was significantly associated with the risk of mortality (*P *< 0.001). Non-survivors had a significantly higher need for mechanical ventilation, renal support and inotropes. Hyperbilirubinaemia > 340 umol/L and coagulation failure (INR > 2.5 or platelets < 20,000) was significantly more often detected in non-survivors. Renal failure and the need for inotropes as a measurement for circulatory failure were significantly associated with mortality on univariate and multivariable analysis. Hepatic encephalopathy was not predictive for mortality. SOFA score and APACHE II on the day of first organ failure predicted risk of death. The AUROC improved for APACHE II and SOFA (Table S4 in Additional file 1). If the patient had a SOFA of 8 or more and this was not improved in a three-day time period their mortality was significantly higher (72.3% vs 46.9%, *P *= 0.013). This failure of improvement over three days had a sensitivity of 75% and a specificity of 48% to predict death. When patients with SOFA of 8 or more were analysed for improvement of SOFA over seven days, failure of improvement had a sensitivity of 74% and a specificity of 61% to predict mortality.

## Discussion

The results of this study support the hypothesis that the occurrence of organ failure in patients with acute deterioration of cirrhosis defines a prognostically and pathophysiologically distinct group. Our data also identify the occurrence of organ failure rather than the severity of liver dysfunction as important factor in determining the prognosis of patients. The demonstration of more than twice the mortality rate in the patients that developed organ failure and had previous decompensation of liver disease illustrates that physiological reserve is important. From a pathophysiological perspective, the data suggest that an altered host response to injury is important in determining the outcome of patients providing the basis of novel prognostic and therapeutic targets.

The outcome of the patients with an episode of decompensated cirrhosis that results in hospital admission is in keeping with the existing previous data. In the group that did not develop organ failure, in-hospital mortality of 8% related mainly to non-liver deaths and a median survival of two years is in keeping with that reported in the literature [13-15]. A survival of 42% in the group that developed organ failure supports the view that attention to the precipitating event and early non-specific supportive management can prevent progression to full-blown multiorgan failure. When patients are admitted to ICU, short-term mortality ranges between 46 and 89% [6,16-31]. These data suggest that the occurrence of a single organ failure in patients with a defined severity of liver disease indicates a poor prognosis. Importantly, both bilirubin and PT were also independent predictors of mortality in keeping with previous data, suggesting that liver and/or associated end-organ failure are associated with poor outcome [6,18,19,21,22,26-29,32-35].

The most important finding of our study was the demonstration that despite a similar precipitating illness and comparable liver function, the patients who had a previous episode of decompensation requiring hospital admission within the previous six months were more than twice as likely to die if they developed organ failure. This observation is akin to the 'P' component of the PIRO concept (Predisposition, Insult/Infection, Response, Organ failure) [7] that has been developed for sepsis-related multiorgan failure. The mechanism underlying the higher mortality rate in the patients with previous decompensation is not clear but is unlikely only to reflect differences in the baseline liver function, since there was no difference in biochemistry and liver function scores between patients with and without previous decompensation. It may well indicate that these patients are more susceptible to injury (I of PIRO) or have an exaggerated inflammatory response with the same precipitating event. Indeed, infection is present in 30% of hospital admissions of cirrhotic patients and the risk of nosocomial infection is nearly six times increased. Mortality of bacteraemia and sepsis in cirrhosis is markedly increased (recently reviewed in [36] and [37]). Infection is closely linked with the occurrence of renal dysfunction, sepsis syndrome and mortality [38-40]. However, in our study, the type of precipitating event had no influence on mortality. One would expect that infection or variceal bleeding causes a higher mortality than other precipitating events. The reason for this might be that some precipitating events lead to organ failure more often, but once organ failure has developed, other factors are more relevant for outcome.

The host response to injury (R) or infection is clinically represented in the occurrence of SIRS. In our study group, we observed SIRS in 42% of patients with organ failure, indicating that pathophysiology of organ failure is similar to that of the sepsis syndrome in which SIRS is crucial in the pathogenesis. However, in our study, SIRS occurred independent of infection - either as a trigger or new infections during the course of illness, indicating that the disease itself might lead to SIRS or that infection is underdiagnosed in patients with ACLF. The presence of SIRS has been shown to be predictive for mortality especially in the subgroup of patients with cirrhosis and renal failure, independent of the presence of infection [41]. SIRS also occurs in about 15% of patients with advanced cirrhosis and acute liver failure and is associated with mortality [42]. In animal models of cirrhosis, administration of lipopolysaccharide (LPS) was associated with a prolonged unremitting inflammatory response, renal dysfunction, encephalopathy and death [43,44]. Although SIRS is present in a significant proportion of patients with organ failure, it is possible that this number is still an underestimation of the true number of SIRS in these patients. The parameter used as SIRS criteria might all be affected by liver cirrhosis - baseline polymorphonuclear count might be reduced due to hypersplenism, baseline heart rate can be elevated because of the hyperdynamic circulatory syndrome, baseline hyperventilation may be present due to hepatic encephalopathy and elevation of body temperature may be blunted in cirrhosis [40]. In our study, we found that inflammation measured CRP at the onset of organ failure was not able to predict of outcome. However, the change in CRP levels over time was able to discriminate between survivors and non-survivors. The poorer outcome in the patients whose CRP levels failed to improve leads us to hypothesise inability to resolve inflammation may be pathophysiologically important in this syndrome. It has recently been shown that decreased HLA-DR expression [45] and a further decrease over three days is noted in non-survivors, whereas survivors showed increased or at least unchanged HLA-DR levels [46].

Our initial hypothesis was that patients with organ failure, who recover, would go back to the clinical state they were in prior to the decompensating event. However, our data clearly show that the patients who recover from organ failure and can be discharged from the hospital have almost universal mortality over the next three years (Figure 2c) suggesting the natural history of cirrhosis is truly altered by the occurrence of organ failure. Similar observations have been made for sepsis [47], where long-term mortality after surviving the index intensive care stay is markedly elevated. So far, it is not fully elucidated, why a survival from an episode of organ failure does not lead to complete recovery. Typically in other patient groups than cirrhosis, acute physiological impairment at admission did not predict long-term mortality, but age - in contrast to our cohort - was predictive in other cohorts.

This study has some limitations: Most patients had alcohol as a major or contributory factor, therefore, these results are most relevant to this patient cohort. However, in most western countries alcohol plays a major role in the pathogenesis of cirrhosis. Another limitation is the fact that we used organ failure scores that were validated in ICU settings also for patients with organ failure on the normal wards. Since there are no organ failure scores for this patient cohort, we believe that it is the best option to use these ICU scores. A third limitation is that we did not prospectively collect biologic material from the whole cohort, therefore, development of novel biomarkers is not possible from this large cohort of patients.

## Conclusions

As has already been described, organ failure is the culmination of several inter-related pathophysiological processes, the presence of which has been described as ACLF. Our data show that although nearly half of the patients can be salvaged if they are developing organ failure, mortality rates become unacceptably high once multiple organ failure becomes established. Our observations confirm that the present organ failure scoring systems can be used to quantify the degree of organ dysfunction but it is possible that at this stage therapeutic options are limited and the main strategy is to prevent progression to multiple organ failure. Clinical and biochemical markers that are able to determine which patients will progress to organ failure following a defined precipitant, is an unmet clinical need. The PIRO system is conceptually useful as it indicates a distinction between the insult and the response. Interventions that target inflammation may impact adversely on the ability to control the infection and interventions that target infection may not be useful if pathophysiological process is being driven through inflammation. The results of this study provide the framework for a better pathophysiological understanding of ACLF taking into account predisposition, injury, response and organ failure which will need to be validated in large, ideally multicentre clinical studies.

## Key messages

• Acute on chronic liver failure is a clinically and pathophysiologically distinct entity characterised by the occurrence of hepatic and extra-hepatic organ failures.

• Progression to organ failure in patients with liver cirrhosis is associated with more severe underlying liver disease and inflammation.

• Previous decompensation, occurrence of new infection, severity of inflammation, lack of its resolution and severity of organ failure are associated with higher mortality.

• The occurrence of organ failure alters the natural history of cirrhosis - nearly all patients die within three years.

• A classification based on the PIRO concept may allow categorisation of patients into distinct pathophysiologic and prognostic groups and allow a multidimensional definition of ACLF.

## Abbreviations

ACLF: acute-on chronic liver failure; ALT: alanine aminotranferase; APACHE II: Acute Physiology, Age and Chronic Health Evaluation; aPTT: activated partial thromboplastin time; CI: confidence interval; CRP: C-reactive protein; ICU: Intensive Care Unit; INR: international normalised ratio; MARS: molecular adsorbent recirculating system; MELD: Model for End-Stage Liver Disease; OF: organ failure; OR: odds ratio; PIRO: Predisposition, Injury, Response, Organ failure; PT: prothrombin time; SIRS: Systemic Inflammatory Response Syndrome; SOFA: Sepsis Organ Failure Assessment.

## Competing interests

The authors declare that they have no competing interests.

## Authors' contributions

RJ designed the study, acquired, analysed and interpreted the results, drafted and revised the manuscript and was responsible for funding. VS designed the study, acquired, analysed and interpreted the results, drafted and revised the manuscript. SS and YMC performed the statistical analysis. LC acquired data and interpreted results. RM supervised the study, acquired, analysed and interpreted the results, drafted and revised the manuscript and provided technical support. All authors read and approved the final manuscript

## Supplementary Material

Additional file 1 **Supplementary data file**. This file contains additional information on data collection and supplementary data tables. Click here for file
