# Supplementary material for: Role of predisposition, injury, response and organ failure in the prognosis of patients with acute-on-chronic liver failure: a prospective cohort study
Source: Crit Care. 2012 Nov 27;16(6):R227. doi: 10.1186/cc11882 (PMC3672612; doi:10.1186/cc11882)
Supplement: Additional file 1 — Supplementary data file. This file contains additional information on data collection and supplementary data tables. [file cc11882-S1.DOCX]

**Supplementary data file**

**Supplementary Methods**

**Data Collection**

Baseline data and mortality were recorded for all patients and an extended dataset including serial data at days 0, 3 and 7 regarding clinical and demographic variables were determined in the organ failure group (Table 2). Age, sex, etiology*,* time from symptom onset to admission, time from admission admission to ICU, time to endpoint (death or discharge from hospital), date of death or date last seen for those patients who were initially discharged, decompensation within the previous 6 months, precipitating event (infection, SBP, variceal bleed, ethanol binge, dehydration, drug induced or other), organ failure within the first week (see definition in the methods section and in Supplementary Table 1), and cause of death. Following clinical parameters were recorded on day 0, day 3 and day 7 in the patients that developed organ failure: sodium, potassium, glucose, urea, creatinine, bilirubin, international normalized ratio (INR), prothrombin time (PT), activated partial thromboplastin time (APTT), albumin, alanin-aminotransferase (ALT), alkaline phosphatase (AP), c-reactive protein (CRP), haemoglobin, hematocrit, white blood cells (WBC), platelets, oxygen saturation, body temperature, mean arterial pressure, heart rate. Severity of liver disease was evaluated by Child-Pugh score and Model for End-Stage Liver Disease (MELD) score at days 0, 3 and 7. Acute Physiology, Age and Chronic Health Evaluation (APACHE) II score was used for determination of illness severity and the Sequential Organ Failure Assessment (SOFA) score for grading of organ dysfunction at days 0, 3, and 7. The presence or absence of SIRS was recorded at days 0, 3 and 7 and the number of failing organs during the first week on ICU was counted (1 point for each: respiratory failure requiring mechanical ventilation, renal failure with a creatinine >221 µmol/L and/or need of haemofiltration, liver failure with a bilirubin over 340 µmol/L according to the calculation of the SOFA score, circulatory failure requiring inotropes and coma (HE 3 or 4). The scores were calculated as published. [[8-12]](#_ENREF_8)

**Supplementary Table S1.** Prediction of mortality is different between groups with and without previous decompensation at the time of diagnosis of first organ failure

| **Previous decompensation** | **AUROC** | **p-value** | **Cut-off** | **Sens.%** | **Spec.%** |
| --- | --- | --- | --- | --- | --- |
| APACHE II | 0.627 | ns | - | - | - |
| SOFA | 0.802 | <0.002 | ≥8.5 | 63 | 83 |
| Child | 0.863 | <0.000 | ≥10.5 | 89 | 56 |
| MELD | 0.802 | <0.002 | ≥9.2 | 82 | 75 |
| **No previous decompensation** | **AUROC** | **p-value** | **Cut-off** | **Sens.%** | **Spec.%** |
| APACHE II | 0.722 | <0.003 | ≥13.5 | 68 | 74 |
| SOFA | 0.706 | <0.007 | ≥9.5 | 50 | 90 |
| Child | 0.846 | <0.000 | ≥10.5 | 93 | 58 |
| MELD | 0.719 | <0.004 | ≥10.7 | 89 | 55 |

**Supplementary Table S2.** Differences between patients with and without an episode of decompensation within the last 6 months at the time of first organ failure

|  | No previous  Decompensation  (N=89) | Previous  Decompensation  (N=70) |
| --- | --- | --- |
| **Predisposition** |  |  |
| Age | 51.8±1.0 | 52.2±1.0 |
| Gender (M/F) | 63/26 | 56/14 |
| Etiology alcohol | 70 (79) | 45 (68) |
| PT (sec) | 18.5±0.7 | 19.5±0.6 |
| INR | 1.73±0.06 | 1.80±0.06 |
| aPTT (sec) | 53.5±2.3** | 66.4±4.0 |
| Bilirubin | 254.8±23.0^**^ | 296.7±18.2 |
| Albumin | 26.2±0.8^*^ | 25.4±0.7 |
| Creatinine | 139.7±14.5 | 156.8±19.5 |
| Child | 11.0±0.2 | 11.5±0.2 |
| MELD | 16.0±1.1 | 17.0±1.4 |
|  |  |  |
| ***Injury (I)*** |  |  |
| Infection n(%) | 45 (50) | 37 (53) |
| Variceal bleed n(%) | 26 (29) | 19 (27) |
| Alcohol binge n(%) | 46 (52) | 41 (59) |
| Dehydration n(%) | 11 (12) | 10 (14) |
| Drugs n(%) | 2 (2) | 1 (1) |
|  |  |  |
| **Response (R)** |  |  |
| SIRS n(%) | 36 (40) | 35 (50) |
| Infection n(%) | 29 (33) | 21 (0.3) |
|  |  |  |
| **Organ failure (O)** |  |  |
| Inotrope | 21 (24) | 26 (37) |
| Renal failure n(%) | 33 (37) | 32 (46) |
| Haemofiltration n(%) | 26 (29) | 19 (27) |
| Mechanical ventilation n(%) | 29 (33) | 26 (37) |
| Hepatic encephalopathy | 51 (57) | 40 (57) |
| Hyperbilirubinemia >340 umol/l | 24 (27) | 24 (34) |
| Severe coagulopathy | 8 (9) | 8 (14) |
| APACHEII | 13.7±0.9 | 13.3±0.9 |
| SOFA | 7.7±0.3* | 9.0±0.4 |

*p<0.05;** p<0.005

**Supplementary Table S3.** Univariate logistic regression analysis of mortality at the time first organ failure is diagnosed stratified by previous decompensation

|  | No previous decompensation | | Previous decompensation | |
| --- | --- | --- | --- | --- |
| Risk factor | OR  (95% CI) | p-value | OR  (95% CI) | p-value |
| **Predisposition** |  |  |  |  |
| Age | 1.00 (0.96, 1.05) | 0.882 | 1.05 (0.98, 1.13) | 0.139 |
| Gender (M/F) | 0.49 (0.19, 1.29) | 0.148 | 4.33 (0.52, 36.17) | 0.176 |
| Aetiology alcohol |  |  |  |  |
| PT (sec) | 1.42 (1.15, 1.76) | 0.001 | 1.44 (1.11, 1.87) | 0.007 |
| INR (per unit) | 5.36 (1.66, 17.34) | 0.005 | 72.4 (4.18, 1251.8) | 0.003 |
| APTT (sec) | 1.03 (1.00, 1.05) | 0.042 | 1.04 (1.00, 1.08) | 0.078 |
| Bilirubin (per 10 umol/L) | 1.03 (1.01, 1.05) | 0.009 | 1.05 (1.01, 1.09) | 0.017 |
| Albumin (g/L) | 0.96 (0.90, 1.02) | 0.190 | 0.92 (0.83, 1.01) | 0.081 |
| Creatinine  (per 10 umol/L) | 1.03 (1.00, 1.07) | 0.085 | 1.05 (0.97, 1.13) | 0.205 |
| Child | 1.76 (1.31, 2.38) | <0.0001 | 2.33 (1.47, 3.70) | <0.0001 |
| MELD | 1.09 (1.03, 1.16) | 0.002 | 1.15 (1.05, 1.25) | 0.003 |
|  |  |  |  |  |
| **Injury** |  |  |  |  |
| Infection | 2.45 (1.03, 5.81) | 0.042 | 1.37 (0.44, 4.31) | 0.589 |
| Variceal bleed | 0.62 (0.24, 1.60) | 0.324 | 0.68 (0.20, 2.34) | 0.544 |
| Alcohol binge | 0.74 (0.32, 1.72) | 0.482 | 0.28 (0.07, 1.10) | 0.068 |
| Dehydration | 2.65 (0.72, 9.83) | 0.144 | 1.11 (0.21, 5.86) | 0.905 |
|  |  |  |  |  |
| **Response** |  |  |  |  |
| SIRS score | 1.69 (1.09, 2.63) | 0.019 | 0.83 (0.47, 1.49) | 0.537 |
| Infection | 6.02 (2.23, 16.27) | <0.0001 | 1.34 (0.37, 4.82) | 0.658 |
| **Organ failure** |  |  |  |  |
| Inotrope | 22.5 (4.76,106.28) | <0.0001 | 12.96 (1.59, 105.90) | 0.017 |
| Renal failure | 7.42 (2.79, 19.79) | <0.0001 | 5.04 (1.27, 20.02) | 0.021 |
| Haemofiltration | 10.62 (3.45, 32.74) | <0.0001 | 3.25 (0.66, 16.07) | 0.148 |
| Mechanical ventilation | 6.18 (0.29, 16.66) | <0.0001 | 5.78 (1.18, 28.25) | 0.030 |
| Hepatic encephalopathy | 0.59 (0.25, 1.39) | 0.231 | 1.71 (0.54, 5.41) | 0.358 |
| Hyperbilirubinemia >340 umol/l | 3.91 (1.45, 10.54) | 0.007 | 4.33 (0.89, 21.11) | 0.070 |
| Severe coagulopathy* | 11.59 (1.36, 99.01) | 0.025 | - | - |
| APACHEII score | 1.16 (1.05, 1.27) | 0.003 | 1.08 (0.97, 1.21) | 0.160 |
| SOFA score | 1.39 (1.14, 1.69) | 0.001 | 1.77 (1.26, 2.49) | 0.001 |

* All 8 patients with an episode of decompensation within the last 6 months at the time of first organ failure died (100% mortality); complete separation of data – logistic regression not performed.

**Supplementary Table S4.** Predictive utility of scoring systems in the study groups

|  | AUROC | p-value | cut-off | Sens.% | Spec.% |
| --- | --- | --- | --- | --- | --- |
| ***Non-organ failure*** |  |  |  |  |  |
| Child | 0.596 | 0.003 | ≥10 | 59 | 56 |
| MELD | 0.626 | 0.000 | ≥16 | 56 | 66 |
| ***Organ failure***  *day 0* |  |  |  |  |  |
| APACHE II | 0.671 | 0.003 | ≥13.5 | 56 | 70 |
| SOFA | 0.741 | 0.000 | ≥9.5 | 48 | 93 |
| Child | 0.830 | 0.000 | ≥10.5 | 90 | 58 |
| MELD | 0.726 | 0.000 | ≥10.7 | 84 | 61 |
| ***Organ failure***  *day 3* |  |  |  |  |  |
| APACHE II | 0.857 | 0.000 | ≥16.5 | 61 | 94 |
| SOFA | 0.876 | 0.000 | ≥9.5 | 72 | 92 |
| SOFA improvement | 0.920 | 0.000 |  |  |  |
| Child | 0.890 | 0.000 | ≥10.5 | 87 | 67 |
| MELD | 0.850 | 0.000 | ≥14.7 | 76 | 81 |

Day 0: The day organ failure is diagnosed

Day 3: 3 days after 1^st^ organ failure is recognised
